# Oral Health-Related quality of life in Portuguese undergraduate students

**DOI:** 10.4317/jced.58810

**Published:** 2021-12-01

**Authors:** Mónica Chantre, Sónia Mendes, Mário Bernardo

**Affiliations:** 1 Graduate Student at Department of Preventive and Community Dentistry Universidade de Lisboa, Faculdade de Medicina Dentária, Unidade de Investigação e Ciências Orais e Biomédicas (UICOB); 2 Professor of Preventive and Community Dentistry. Universidade de Lisboa, Faculdade de Medicina Dentária, Unidade de Investigação e Ciências Orais e Biomédicas (UICOB)

## Abstract

**Background:**

The Oral Health-related Quality of Life (OHRQoL) is an essential part of health and wellbeing that aims to assess the impact of oral health on aspects of personal and social life. This investigation aimed to study the OHRQoL in undergraduate students and relate it to sociodemographic, academic behaviours, oral health behaviours, the presence of oral problems and self-perception of oral health.

**Material and Methods:**

The study target-population consisted of the undergraduate students attending the University of Lisbon (UL). Data collection was carried out through an online questionnaire which included self-reported sociodemographic and academic characteristics, behaviours and oral health status, and the Oral Health Impact Profile-14 (OHIP-14). Descriptive statistics were performed, and the Mann-Whitney and Kruskal-Wallis tests were used (α=0.05).

**Results:**

The sample included 933 students, aged between 18 and 48 years old (mean=21.22 / SD=3.11). The global mean value of OHIP-14 was 5.98 (SD=6.71) and 89.8% of the students presented OHIP-14 values between 0 and 14. Psychological discomfort and physical pain were the dimensions of OHIP-14 with the greatest impact on OHRQoL. Most of the students brushed their teeth twice a day (79.7%) with fluoridated toothpaste (90.8%) and perceived their oral health as “good” (56.3%). Several aspects were significantly related (*p*<0.05) to a worse OHRQoL, namely, being of African origin, courses not related to health, changes for worse in oral hygiene habits after entering university, higher consumption frequency of cariogenic foods or, going to oral health appointments in urgent situations, not having oral health appointments for economic reasons, history of oral health problems, self-reported oral problems and negative self-perception of oral health status.

**Conclusions:**

Most UL students had a good OHRQoL, adequate oral health behaviours and a good self-reported state of oral health.

** Key words:**Oral health-related quality of life, OHIP-14, Oral health behaviours, Self-reported oral health, University students.

## Introduction

The assessment of oral health using exclusively clinical criteria makes it impossible to analyse the impact of oral problems on life and general well-being. Quality of life is recognized as being a valid parameter, used for evaluation in different areas of health, including oral health, making it possible to consider the impact of oral health on aspects of the individual’s personal and daily life (1,2). Quality of life corresponds to the person’s set of perceptions about his/her position in life, in the cultural context and the value systems present in the society and in relation to one’s goals, expectations, standards and concerns (2).

The evaluation of Oral Health-Related Quality of Life (OHRQoL) main objective is to determine the psychosocial effects resulting from the oral health state (2). OHRQoL reflects the general well-being of the individual in common aspects of daily life, such as speaking, eating, sleeping, as well as satisfaction with oral health, self-esteem, academic and professional performance, and social interaction (2,3). The multidimensionality of OHRQoL fits into the biopsychosocial model of health, including factors such as socioeconomic status, oral health status, self-perception of oral health and health-related psychological factors (4).

Despite being a relatively recent concept, OHRQoL is considered a valuable health indicator, with important implications for the clinical practice, research, and education in oral health (2,5). The recognition of the importance of psychosocial health indicators led to the development of instruments to quantify OHRQoL. One of the most used instruments, due to its psychometric qualities and ability to measure self-perception of the consequences related to oral diseases, is the “Oral Health Impact Proﬁle” (OHIP) (1,3,6,7).

The first version of the OHIP, developed and validated by Slade in 1994 (7). It was developed as a comprehensive measure of self-reported dysfunction, discomfort, and disability of oral conditions, to complement conventional oral epidemiological indicators, providing information on the impact of oral diseases on populations and the effectiveness of health services in reducing that impact (7). The OHIP is based on Locker’s oral health model, which encompasses the three dimensions of oral health (physical, psychological, and social) distributed over seven dimensions of quality of life, namely, functional limitation, physical pain, psychological discomfort, physical disability, psychological disability, social disability, and handicap (5,7). The 1994 version consists of 49 items (OHIP-49) distributed over the seven dimensions. In 1997, Slade developed, through regression analysis of the original index, a new simplified version with 14 items (OHIP-14), which includes two items for each of the instrument’s seven dimensions (8).

Several studies on OHRQoL have been carried out in young adults, including university students (1,3,6). University students go through a period of growth and development, characterized by the dynamic transition between adolescence and adulthood (3). Entering higher education is characterized by various psychosocial challenges which are heightened when students must leave their home environment. For the first time in their lives, in most instances, they must face the separation from family and friends, face a new reality and become more independent in their choices, which can lead to changes in lifestyle and behaviours with health implications (3,9).

When negative changes occur in oral health behaviours, the clinical status tends to worsen. Inadequate oral health behaviours, such as a high consumption of carbohydrates or inappropriate toothbrushing habits, can lead to adverse effects on oral health and, consequently, on OHRQoL (3,10-14).

According to some studies, university students with better self-perception of their oral health, clinical status, and oral health behaviours, as well as fewer subjective oral symptoms, report better OHRQoL (3,6). It is also expected that those studying in health areas are more aware of health-related problems, including oral health. In addition, these students tend to have a higher socioeconomic level, as well as greater health literacy, which, in turn, can lead to a better self-assessment of their oral health and, ultimately, to a better OHRQoL (6). Many studies show the existence of a relationship between poor oral health and a worse quality of life (1,3,15).

This study aims to assess the OHRQoL of undergraduate students attending the University of Lisbon and its relation to sociodemographic and academic characteristics, oral health behaviours, self-reported oral conditions, and self-perception of their oral health status.

## Material and Methods

To achieve the proposed objectives, a cross-sectional study was carried out on undergraduate students attending the University of Lisbon, during the academic year 2019/20. The study was approved by the Health Ethics Committee of the Faculty of Dental Medicine of the University of Lisbon.

Data collection was performed by completing an online questionnaire. On the questionnaire’s homepage, all information related to the study was presented, including its objectives, procedures, and explanation that participation was voluntary and anonymous.

The questionnaire was developed based on a literature review (1,3,13,16), and consisted of thirty questions directed to the collection of sociodemographic information, behaviours, self-perception of oral health and OHRQoL. To study this last variable, the Portuguese version of OHIP-14 (16) was used. The OHIP-14 score is calculated by adding the points from all the items, and varies between 0 and 56, with the highest values corresponding to a worse OHRQoL (7,17).

The distribution of the questionnaire was carried out through the student’s associations of the University of Lisbon. The questionnaire was available between January and June 2020.

Statistical analysis was performed using the IBM SPSS Statistics software (version 26). Mann-Whitney and Kruskal-Wallis tests were used, with a significance level of 5%.

## Results

The study sample consisted of 933 participants, with a mean age of 21.22 years (sd=3.11). Most participants were female (80.5%) and all the schools of the University of Lisbon that have graduation courses were represented.

**Figure 1:**
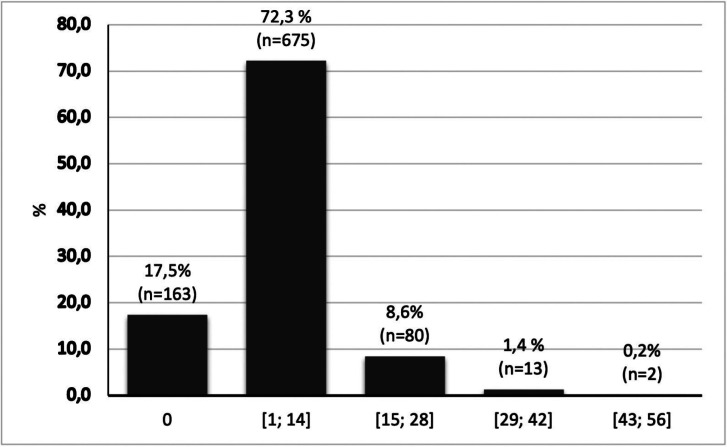
Distribution of OHIP-14 values.

The mean global value of the OHIP-14 was 5.98 (SD = 6.71), with a minimum of 0 and a maximum of 53. Most of the students (89.8%) were in the lowest range of OHIP-14 values [0-14] and only 1.6% were in the highest range [29-56], corresponding to a worse OHRQoL (Fig. 1).

Table 1 shows the frequency of responses and the mean values for each of OHIP-14 items, grouped by their dimensions.

**Table 1:**
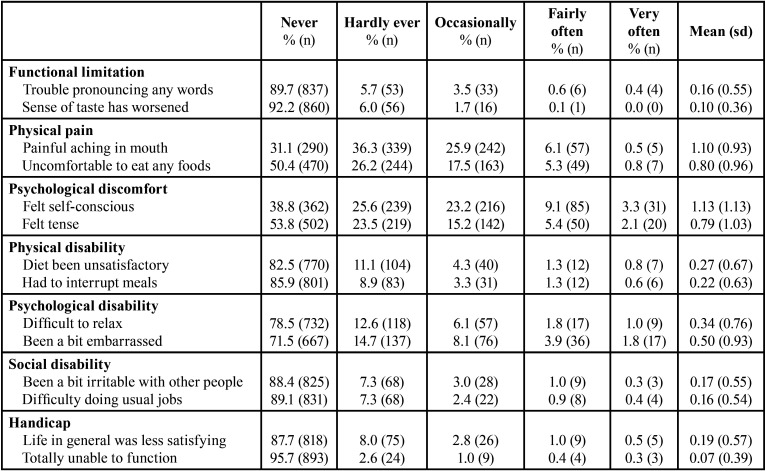
Frequencies, means and standard deviations of OHIP-14 items.

There were statistically significant differences in relation to the region of origin (*p*=0.001) and area of the course (*p*=0.002), with students of African origin and those belonging to courses not related to health having a higher OHIP-14 score (Table 2).

**Table 2:**
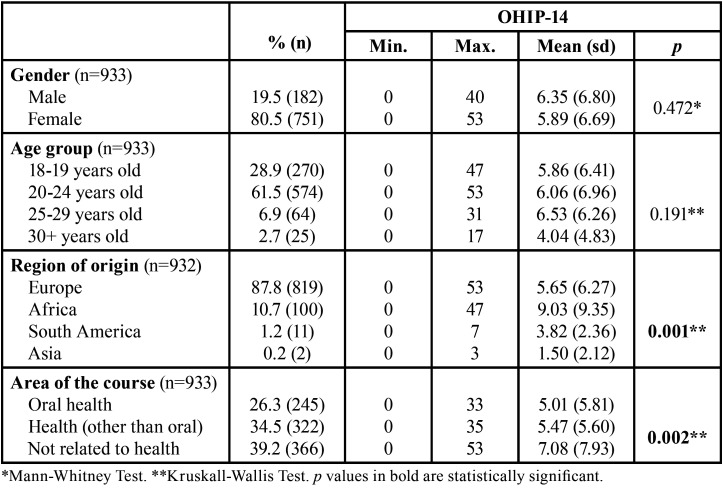
OHIP-14 distribution according to sociodemographic and academic characteristics.

The change in oral hygiene habits after entering university, the cariogenic food frequency of consumption, and the reason for having or not having an oral health appointment in the last year, were significantly associated with OHRQL (*p*<0.001) (Table 3).

**Table 3:**
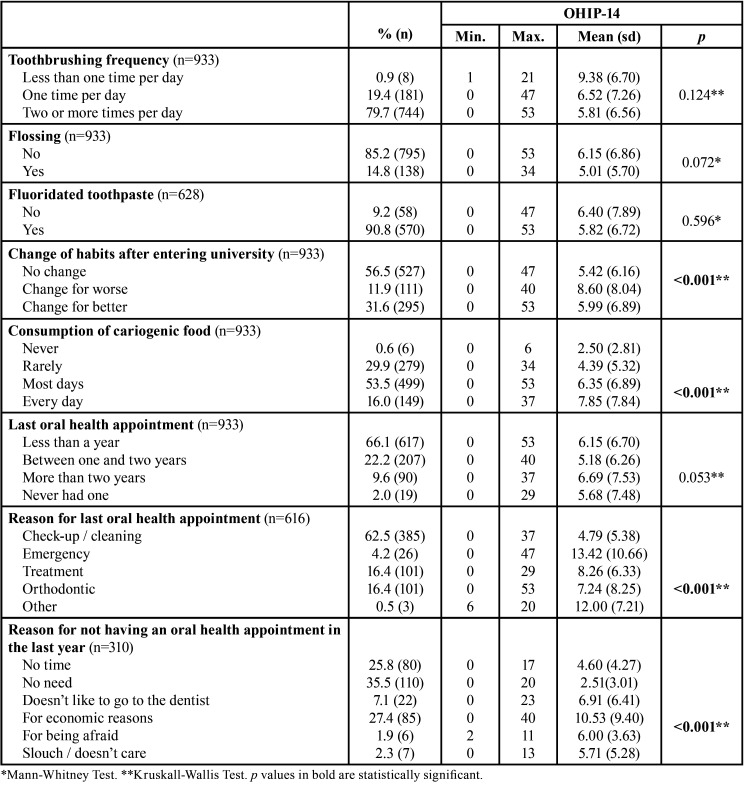
OHIP-14 distribution according to oral health behaviours.

As can be seen in Table 4, all self-reported oral health conditions and self-perceived oral health status were significantly associated with OHRQoL (*p*<0.05).

**Table 4:**
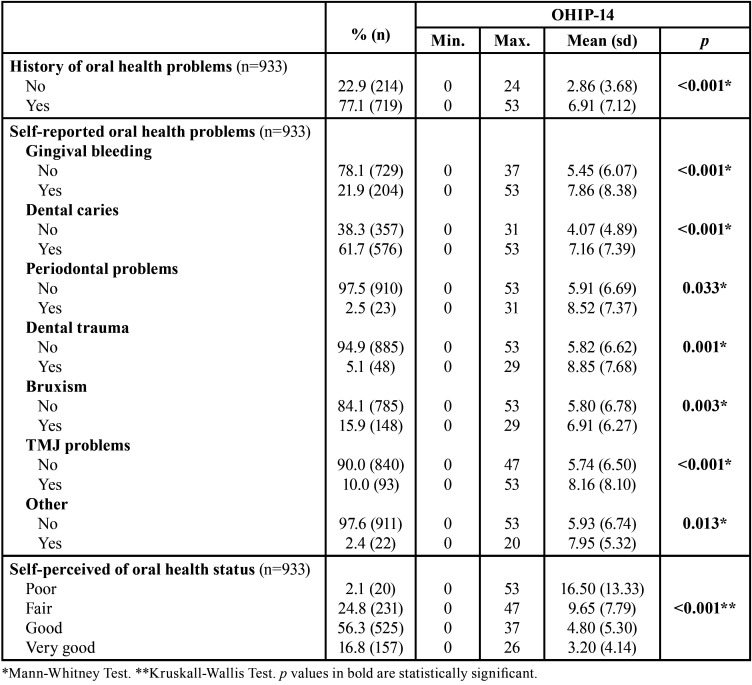
OHIP-14 distribution according to self-reported oral health problems and self-perceived oral health status.

## Discussion

The OHRQL of university students is a topic scarcely studied in Portugal. The study sample consisted of 933 students, corresponding to 2.65% of the total 35261 undergraduate students attending the University of Lisbon.

In the present study, the average global OHIP-14 value was 5.98. This value is higher than the 1.92 obtained by Yamane-Takeuchi *et al*. (3) and the 4.63 by Drachev *et al*. (1), performed respectively on Japanese and Russian university students. It was also higher than the 3.3 obtained in the study by Montero *et al*. (10), carried out in a sample of Portuguese adolescents aged between 11 and 17 years. The disparities found between the studies may be related to the different sociodemographic characteristics of the studied samples.

Most students in the present study (89.8%) were in the lower range [0-14] of the OHIP-14 scores, with 17.5% having a value equal to zero. These results reveal a good OHRQoL in the studied population. However, 82.5% of the sample had at least one impact in one of the OHIP-14 items, a result higher than the one obtained in a sample of Norwegian adults (65%) (4). This value was also higher than in a study carried out in Portuguese adolescents (56.3%) (10). Psychological discomfort and physical pain were the dimensions of OHIP-14 that revealed the greatest impact on OHRQoL, a result similar to several other studies (1,3,4).

Some studies have reported that sociodemographic characteristics, such as socioeconomic status, region of origin, area of residence, level of education and course area are associated with oral health status and quality of life (1,2,4,6,18). According to the literature, male and younger individuals have better OHRQoL, compared to female and older individuals (4). However, in the present study, there were no significant differences between genders or age. The only sociodemographic variable for which significant differences were found was the region of origin, with students from Africa being those who presented worse OHIP-14 scores, highlighting that it is important to take cultural characteristics into account.

Regarding academic features, it was found, like in other studies (1,6,12,15), that students of the health and the oral health courses had better OHRQoL compared to students enrolled in courses unrelated to health. In the present study, oral health students revealed values that indicate a better OHRQoL, which would be expected as these students have better knowledges, attitudes and behaviours related to oral health, as well as better access to dental care, which may have a direct impact on OHRQoL (1,6).

It is interesting to note that most of the students reported to brush their teeth twice a day with a fluoride toothpaste, as well as having an oral health appointment in the last year for “check-up/cleaning”. These results show a satisfactory implementation of healthy habits. Twice a day toothbrushing (79.7%) was found to be higher than the 65% obtained in the study by Montero *et al*. (10), carried out in a sample of young Portuguese individuals aged between 11 and 17 years. These differences may result from the age difference between the samples, cultural differences, and from the level of knowledge about oral health behaviours acquired along the academic path (1,4). In contrast, the frequency of toothbrushing twice a day was lower than the 86.7% found by Yamane-Takeuchi *et al*. (3), in Japanese university students.

The low frequency of flossing and frequent consumption of cariogenic food were the worst behaviours identified in the study population.

Students who reported negative changes in oral health habits after entering the university, showed a worse OHRQoL. These results are in accordance with many other studies (3,13,14) and suggest that healthy behaviours are associated with better OHRQoL. Montero *et al*. (10) and Yamane-Takeuchi *et al*. (3) found that poor oral health behaviours were associated with a worse OHRQoL. In the present study it was also found that students who consumed cariogenic food every day have a worse OHRQoL.

Additionally, students that did not visit the dentist in the previous year for economic reasons had a worse OHRQoL. The socioeconomic level has an impact on access to health care, resulting in disparities in oral health (2). Students with better socioeconomic status tend to seek oral health services more frequently and, when associated with good oral hygiene habits, they tend to be more satisfied with their oral health status.

In the present study, most students reported the presence of oral health problems. Like other studies, dental caries and gingivitis were the most reported oral health problems (10,19). History of dental trauma was related to the worst OHIP-14 score. In addition to functional limitation, aesthetic damage and treatment costs, dental trauma has also a psychosocial impact, especially in young persons (20). The second oral health problem with the greatest impact on OHRQoL was periodontal disease. These results are similar to those of other studies carried out on samples of young adults, in which a negative impact of periodontal diseases on OHRQoL was observed (21).

Regarding self-perceived oral health, it was found that most students classified their oral health status as “good” or “very good”, with these individuals presenting a better OHRQoL. Several studies, carried out on university students from different countries, suggest the hypothesis that poor oral health is correlated with poorer self-perception and with worse OHRQoL (1,3,6).

## Conclusions

In the present study, most of the students showed a good OHRQoL, and their oral health behaviours were very satisfactory. Several aspects such as the course area, the region of origin, the change in oral hygiene habits after entering the university, the frequent consumption of cariogenic foods, and the search for oral health care proved to be important factors to consider regarding OHRQoL in university students.
